# Health policies and the relationships between socioeconomic status, access to health care, and health

**DOI:** 10.1186/2045-4015-2-50

**Published:** 2013-12-19

**Authors:** Bénédicte H Apouey

**Affiliations:** 1 Paris School of Economics – CNRS, 48, Boulevard Jourdan, 75014 Paris, France

## Abstract

Health policies tend to focus on improving the access to health care of persons of low-socioeconomic status to improve their health. This commentary argues that health policies directly directed at health and socioeconomic status (and other components of individual welfare) will also be effective if one wants to improve the well-being of the poor.

## Introduction

In an illuminating paper, Joseph Deutsch, Adi Lazar and Jacques Silber investigate whether individuals facing the threat of poverty curtail their consumptions of various goods and services, including health expenditures, in a given order [1]. They take a multidimensional approach to poverty measurement, as they assume that individual deprivation depends on up to seven goods that individuals gave up over the previous year because they could not afford them^a^. Using the 2003 Israeli Social Survey, they show that dental work is one of the first expenditures that people cut back on when facing the risk of becoming poor. People also cut back on prescription drugs, additional health insurance, and other medical treatments, albeit to a lesser extent. Moreover, using information on the stage of individuals in the order of curtailments, the authors construct an individual deprivation index, to examine the determinants of deprivation. They find that individuals in their late 30′s and early 40′s, with children under 5, in poor health, from large households, with a low educational level, and a low income, tend to experience a significantly greater level of deprivation.

Thus, the article by Deutsch and colleagues examines the relationships between poverty, socio-demographic characteristics, and health expenditures. This focus stems from a more fundamental concern about the health of persons of low socioeconomic status (SES, i.e. income, wealth, education...). A very substantial literature in social sciences has shown that higher SES is associated with better health [2,3]. However, this relationship is still not perfectly understood. Deutsch and colleagues emphasize several interpretations of this relationship, by showing that individuals who face the risk of becoming poor cut back on health expenditures, and that health, income, and education are predictors of financial deprivation. In this brief commentary, I discuss how understanding the mechanisms that relate socioeconomic status and health could help design sound health policies.

### From socioeconomic status to access to health care and health

The underlying assumption of the article regarding the relationship between SES, health care and health is that high-SES individuals have better access to health care, which has a positive effect on health^b^. Indeed, high-SES individuals may have better access to health care services, thanks to their greater income. One would expect that income has an effect on access to health care for health care services that are not covered by basic coverage packages in countries with universal health coverage (like dental work in Israel, which is analysed in the paper), and for all types of health care services in countries where health care coverage is not universal and where the market has a large role in the financing of care (like the US). In addition, in all countries (with and without universal health coverage), high-SES people may have access to better quality health care and be more compliant with therapies, so that medical care may be more effective for them [4]. From a policy perspective, this interpretation of the SES/health relationship implies that policies should improve access to health care, adherence to treatments, and the quality of care for persons with low-SES.

The empirical literature shows that there is a causal effect of SES on access to health care. For instance, Kim and Ruhm [5] found a significant impact of (exogenous) income shocks on out of pocket expenditures and - the probability of - dental care, using data on older Americans [5]. However, both health economics and public health research indicate that access to health care does not provide a complete explanation of the SES/health gradient. In particular, the existence of large social health inequalities in countries with free health care at the point of service (like the UK) shows that the relationship between SES and health is not primarily explained by differences in access to health care [4]. As such, policies that only pay attention to access to health care may not be adequate.

### From socioeconomic status to health

Leaving aside access to health care, the positive correlation between SES and health may be interpreted in three different manners. First, SES may have an impact on health. This effect could operate through factors such as nutrition and housing conditions. The empirical literature finds that there is a small but significant effects of SES on health in Germany, the UK and the US, using unanticipated (exogenous) shocks in income [5-7]. This mechanism implies that income redistribution toward the poor could help improve their health.

Second, health may determine SES, through physical and mental capacity for work. This second interpretation is developed by Deutsch and colleagues, when they highlight that health is a predictor of poverty. This channel means that a sound policy will try to directly improve the health of individuals (and not just improve access to health care).

Third, the correlation between SES and health could be spurious and arise because of the omission of common hidden factors. Early life conditions are a good example of such hidden factors, since the early life conditions could be effecting both SES and health in adulthood [8,9]. In that case, policies need to address these hidden factors in order to improve both SES and health.

Obviously, the three mechanisms may operate simultaneously to explain the correlation between SES and health.

## Conclusion

Identifying the most relevant mechanisms that underlie the correlations between socioeconomic status, health and utilization of health care is a great avenue for future research and it will contribute to efforts to develop appropriate health policies. Current knowledge tends to emphasize the need for policies directly targeted at SES and health, rather than access to health care only. In doing so, the goal of policy makers should be to improve the well-being of the poor, which is multidimensional by definition.

Deutsch and colleagues already take a multidimensional approach to the measurement of poverty as they account for several components of welfare. This is a very relevant contribution to the health economics literature that is generally uni- or bi-dimensional. In a very general setting, individual well-being will include SES (income, wealth, education…), health and other components. Because of this multidimensionality, policy makers should be cautious about not improving SES at the expense of health or other components, or the contrary [4].

## Endnotes

^a^Specifically, they consider up to seven goods from the following list: clothing or shoes; dental work; heating or cooling; food; prescription drugs; electricity or phone; another medical treatment.

^b^See for instance the two last sentences in the conclusion of the paper: “[…] the cutback in health expenditures is an important signal of the impoverishment process. Such a conclusion should have important policy implications if one accepts the principle according to which the right to a decent health should be one of the basic human rights”.

## Competing interests

The author declares that she has no competing interests.

## Authors’ information

BA is a research professor at the Paris School of Economics - CNRS.

## Commentary on

Deutsch J, Lazar A, Silber J. Becoming poor and the cutback in the demand for health services in Israel. Isr. J. Health Policy Res 2013, 2:98.
